# Improving the Training Experience of International Medical Graduates (Imgs): A Survey of Psychiatry Trainees in the Yorkshire & Humber Deanery (West/East/North)

**DOI:** 10.1192/bjo.2022.290

**Published:** 2022-06-20

**Authors:** Christiana Elisha-Aboh, Ogba Onwuchekwa, Rahul Watts, Anilkumar Pillai, Sara Davies

**Affiliations:** 1 Leeds and York Partnership NHS Foundation Trust, UK, Leeds, United Kingdom; 2 Tees Esk & Wear Valley NHS Foundation Trust, UK, York, United Kingdom; 3 Bradford District Care NHS Foundation Trust, UK, Bradford, United Kingdom; 4 Old Age Training Programme Director, Health Education England Yorkshire & Humber, Bradford, United Kingdom; 5 General Adult Training Programme Director, Health Education England Yorkshire & Humber, Halifax, United Kingdom

## Abstract

**Aims:**

There are over 72 000 licensed IMGs in the UK who fill up crucial shortages in the NHS and provide diversity. In 2020 there were more IMGs than local graduates joining the General Medical Council register with over half (54%) identifying as Black and Minority Ethnic doctors. There are ongoing and extensive conversations about the best approach to tackle differential attainment between IMGs and local graduates. The aims were to identify what the perceived differences were between local graduates and IMGs in various domains and recognise what measures could be taken to improve the issues identified.

**Methods:**

This survey utilised the Typeform survey software to ask 23 questions and was left open for 3 months. Participation in the survey was voluntary and anonymized and included feedback from both Core Trainees and Higher Trainees. Initial emails, texts and chats with the survey link and reminders were sent to the Medical Education departments and trainee groups. The qualitative and quantitative data from all 33 respondents were analysed.

**Results:**

90.9% (30) of participants felt there were issues of differential attainment between IMGs and local graduates and felt that the gaps in differential attainment could be addressed by mentoring, networking, IMG lead roles, education of trainers and better support systems. 57.6% (19) of IMGs stated that they had felt bullied, undermined, treated unfairly, or intimidated; with only 29% (9) attempting to challenge this due to the fear of retribution, concerns about accountable, cultural and communication barriers. All respondents felt induction programmes, focusing on IMGs and cultural diversity would be helpful for all trainees, with 93.9% (31) of respondents recommending that more education was needed for trainers. 57.6% (19) stated that they had considered relocating outside the UK after training because they felt they would be better valued elsewhere. 90.9% (30) suggested that a book for IMGs would be a welcomed development. 87.9% (29) recommended that having IMG leads was important for offering well-being support, play a safeguarding role, offer pastoral care, and contribute to induction and education; with 68.8% (22) recommending the person was a College trainer.

**Conclusion:**

These findings highlight several challenges IMGs training in the UK face and must navigate to be successful. A greater awareness of their hurdles is critical to maximising what potentials lie within. As the numbers of IMGs within the system continue to rise, there is an even greater need to support and address the concerns this survey underscores.

